# De Novo *RRAS2* Pathogenic Variant in a Fetus With Bilateral Radial Ray and Multisystem Anomalies

**DOI:** 10.1002/pd.70137

**Published:** 2026-03-20

**Authors:** Nicole R. Legro, Lylach Haizler‐Cohen, William Genthe, Victoria Greenberg

**Affiliations:** ^1^ Department of Obstetrics and Gynecology Division of Maternal‐Fetal Medicine MedStar Washington Hospital Center Washington District of Columbia USA; ^2^ Children's Mercy Kansas City Missouri USA; ^3^ Georgetown University School of Medicine Washington District of Columbia USA

**Keywords:** Noonan syndrome, radial aplasia, rasopathy, *RRAS2*

## 1 Fetal Phenotype

The patient was a 30‐year‐old G2P1001 with a past medical history of Lyme meningitis and prior normal spontaneous vaginal delivery. Her son was born large for gestational age and had hypospadias at birth but otherwise developed typically. Screening for hemoglobinopathies, cystic fibrosis, and spinal muscular atrophy was normal. The patient was of Nicaraguan, German and French ancestry, while her 35‐year‐old partner was of German, Dutch, Irish, and Ashkenazi Jewish ancestry. Obstetric and family history were non‐contributory (Table 1A).

**TABLE 1A:** Clinical data.

Case	Parental details			Gestation at diagnosis	Phenotypes (HPO terms)	Obstetric history	Family history	Outcome
1	Maternal	Age	30	20 weeks	Echogenic intracardiac focus (EIF) (HP:0010942) Prominent right pulmonary vein/venous abnormality (HP:0002624) Ventricular septal defect (VSD) (HP:0001629) Macrosomia (EFW > 99%, 549g at 20w6d) (HP:0001520) Bilateral radial aplasia (HP:0004977) Broad and curved thumb (HP:0011304) Clinodactyly of 2nd digit (HP:0040022) Scoliosis (Congenital) (HP:0002650) Limb undergrowth (FL/AC < 1%) (HP:0009826) Lumbar hemivertebrae (HP:0008439) Sacral hemivertebrae (HP:0002937) Bilateral pylectasis (HP:0011129) Echogenic kidneys (HP:0004719) Megacystis (HP: 0010956) Single umbilical artery (HP:0001195) Abnormal placental membrane morphology (placental lakes) (HP:0011409)	LGA	None	Termination
		Ethnicity	Nicaraguan, German, French					
	Paternal	Age	35					
		Ethnicity	German, Dutch, Irish, ashkenazi jewish					

Abbreviation: LGA, large for gestational age.

**FIGURE 1:**
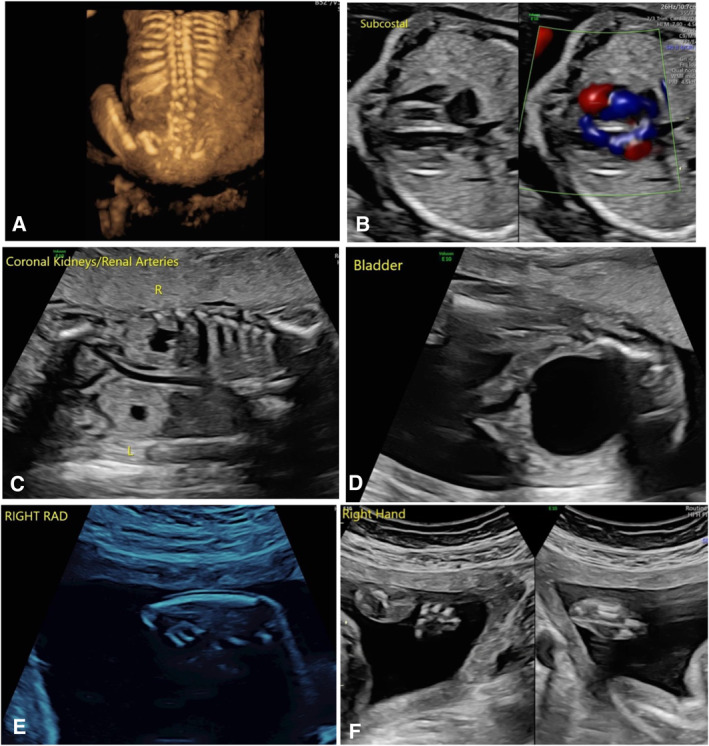
Prenatal ultrasound findings at 20 weeks' gestation. (A) Lumbar hemivertebra. (B) Ventricular septal defect, subcostal view. (C) Dilated renal pelvis bilaterally, left renal pelvis 8.7 mm, right 6.1 mm (measurement not shown). (D) Enlarged fetal bladder and single umbilical artery. (E) Single right forearm bone and radial curvature of the right hand. (F) Right hand.

She was referred to Maternal Fetal Medicine at 20 weeks of gestation. A detailed anatomy survey by MFM identified a male fetus with estimated fetal weight > 99% (549 g). Fetal anomalies included a ventricular septal defect and a prominent right pulmonary vein, suggestive of a possible venous abnormality. Genitourinary anomalies included megacystis and bilateral renal pelvis dilation. Skeletal findings were particularly striking, including lumbar hemivertebra and only one forearm bone bilaterally, raising suspicion for bilateral absence of the radii. There was radial curvature of both hands, a broad and curved left thumb, and clinodactyly of the second digit on the right hand (Table 1A, Figure 1A–F).

## 2 Diagnostic Method

Amniocentesis was performed at 22 weeks' gestation. Both karyotype and chromosomal microarray analyses were normal. Trio exome sequencing was perfomed by direct analysis (uncultured cells) using the Ambry Genetics Exome Next test (Table 1B). There were no secondary findings or variants of uncertain significance. The variant was interpreted according to the American College of Medical Genetics and Association for Molecular Pathology criteria.

**TABLE 1B:** Clinical and genetic findings.

Procedure	Direct or culture	Test	Gene	Known disease (OMIM)	Variant	ACMG classification	Criteria applied	Inheritance	Interpretation	ClinVar accession number
Amniocentesis (22 weeks)	Direct	Trio exome	*RRAS2* (NM_012250.6)	*RRAS2*‐related Noonan syndrome (618624)	Heterozygous c.68G>T p.(Gly23Val)	Pathogenic	PS2, PS3, PM2_supporting	Autosomal dominant, de novo	Pathogenic	RCV000852396.2

Abbreviations: M, maternal; P, paternal.

## 3 Diagnostic Results and Interpretation

Results showed a de novo gain‐of‐function pathogenic variant in *RRAS2*: c.68G>T, p.(Gly23Val) associated with Noonan Syndrome. Previously reported functional analysis of this variant showed a gain‐of‐function mechanism consistent with most Noonan‐syndrome‐related genes [1].

## 4 Pregnancy Outcomes

The couple elected to pursue termination of pregnancy due to the severity of the anomalies and the anticipated poor prognosis. They declined an autopsy.

## 5 Discussion

*RRAS2* gain‐of‐function variants have been linked to classical Noonan syndrome with prenatal manifestations including macrosomia, polyhydramnios, ventricular septal defect, facial anomalies and anal atresia (Table S1) [2]. The c.68G>T, p.(Gly23Val) variant has been described in two previous cases, both in males and occurring de novo [1, 3]. These individuals had developmental delays and intracranial abnormalities, including Chiari I malformation and arachnoid cysts. One of the probands was reported to have a “broad thumb” but no other skeletal features were found [1, 3]. In the broader *RRAS2‐*related Noonan syndrome cohort, reported limb anomalies include pes planus and proximally‐placed thumbs, while other skeletal findings such as pectus excavatum and spinal canal stenosis have been described [1, 2, 3]. A prenatal cohort analysis of pregnancies with germline pathogenic variants in the RAS‐MAPK pathway identified limb anomalies limited to syndactyly, clubbed feet, and short femurs [4]. The presence of a single forearm bone has not been previously documented in association with *RRAS2* pathogenic variants.

Genitourinary anomalies have been reported in at least four cases of pathogenic *RRAS2* variants. Prior findings include cryptorchidism, bilateral hydronephrosis, unilateral duplex kidney, hypoplastic scrotum, testicular hydrocele, and micropenis [1, 2, 3]. Megacystitis, as in this case was not previously reported in association with an *RRAS2* variant and may reflect an outlet obstruction.

This case presents several possibilities: a possible phenotypic expansion of the skeletal manifestations of *RRAS2*‐related Noonan syndrome, severe radioulnar synostosis, or a dual diagnosis. Absence of the bilateral radii raises the possibility of a radial ray anomaly, with a differential including Holt–Oram syndrome, Fanconi anemia, thrombocytopenia‐absent radius syndrome, amongst other environmental causes such as diabetic embryopathy [5]. However, these conditions were not identified on phenotype‐driven exome sequencing and diabetes screening was negative. The anomalies in this fetus raise the possibility of a concurrent VACTERL association or an additional pathogenic variant not detected by current genomic technology. Radioulnar synostosis has previously been described with Noonan syndrome, which is in contrast to the apparent absence of the radius in this case [6].

Genetic counseling included discussion of germline mosaicism, recurrence risk, and options for prenatal diagnostic testing in future pregnancies. This case underscores the challenges of prenatal phenotyping while illustrating opportunities to expand the known spectrum of genetic diseases.

## Funding

The authors have nothing to report.

## Ethics Statement

The authors have nothing to report.

## Consent

Consent was obtained from the study participant.

## Conflicts of Interest

The authors have reported all conflicts of interest.

## Supporting information


**Table S1:** Previously reported pathogenic and likely pathogenic variants in *RRAS2* ClinVar database.

## Data Availability

Data pertaining to this clinical case is limited by privacy and available upon request by contacting the corresponding author.
