# Supplementary material for: De Novo RRAS2 Pathogenic Variant in a Fetus With Bilateral Radial Ray and Multisystem Anomalies
Source: Prenat Diagn. 2026 Mar 20;46(4):603–6. doi: 10.1002/pd.70137 (PMC13070214; doi:10.1002/pd.70137)
Supplement: Supplementary file 1 — Table S1: Previously reported pathogenic and likely pathogenic variants in RRAS2 ClinVar database. [file PD-46-603-s001.docx]

| ***RRAS2* variant and predicted protein change** | **ClinVar classification** | **Phenotype** | **PMID** |
| --- | --- | --- | --- |
| c.68C>T p.Gly23Val | Pathogenic | As reported in this publication and Iida et al. and Capri et al. | 38601074  31130282 |
| c.68G>C p.Gly23Ala | Pathogenic | NR | N/A |
| c.68G>A p.Gly23Asp | Pathogenic | Noonan syndrome 12 | 35599849 |
| c.71G>A p.Gly24Asp | Pathogenic/Likely pathogenic | Noonan syndrome 12 | N/A |
| c.67G>T p.Gly23Cys | Pathogenic/Likely pathogenic | Noonan syndrome: Prominent forehead, Hypertelorism, Hydrocephalus, Rhabdomyosarcoma, Short stature | 37942564 |
| c.208G>A p.Ala70Thr | Likely pathogenic | Noonan syndrome: Mild facial anomalies, VSD, strabismus, unilateral duplex kidney | 31130282  35979676 |
| c.212G>A p.Gly71Glu | Likely pathogenic | Noonan syndrome: Facial features, patent foramen ovale, moderate global delay, myelin development delay, subarachnoid width | 36460282 |
| c.215A>T p.Gln72Leu | Pathogenic | Noonan syndrome: Nuchal edema, fetal ventriculo-megaly and cardiac abnormalities, hypoplastic scrotum, tetralogy of Fallot, dilated cardiomyopathy, pectus excavatum, intellectual disability, hydrocephalus, 11 rib pairs, proximally placed thumb, spinal canal stenosis, thrombocytopenia, labyrinth dysplasia, anteriorly placed anus, cryptorchidism | 31130282  31130285 |
| c.62_73dup p.Gly21_Gly24dup | Likely pathogenic | NR | N/A |
| c.70_78dup p.Gly24_Gly26dup | Pathogenic | Noonan syndrome 12  Abnormal pinna morphology, Micrognathia, Abnormality of the face, Polyhydramnios, Ventricular septal defect, Hypotonia, Femur fracture, Ventricular septal defect, Pulmonic Stenosis, Congenital laryngomalacia, Rib fusion, Nasolacrimal duct obstruction, Myopia | 31130282  31130285 |
| c.65_73dup p.Gly22_Gly24dup | Pathogenic | Noonan Syndrome: Nuchal edema, polyhydramnios, poor feeding, supravalvular aortic stenosis, glabellar heamangioma | 31130282 |

Supplemental table 1. Previously reported pathogenic and likely pathogenic variants in *RRAS2* ClinVar database.
